# Resistance Analysis of Crack Propagation in Concrete Subjected to Hydraulic Pressure

**DOI:** 10.3390/ma17133243

**Published:** 2024-07-02

**Authors:** Yang Wang, Jingshan Sun, Gaohui Wang, Yongzhen Li, Weiqi Xiong

**Affiliations:** 1 State Key Laboratory of Precision Blasting, Jianghan University, Wuhan 430056, China; whuwangyang@whu.edu.cn; 2 Hubei Key Laboratory of Blasting Engineering, Jianghan University, Wuhan 430056, China; 3 School of Water Resources and Hydropower Engineering, Wuhan University, Wuhan 430072, China

**Keywords:** hydraulic fracturing, concrete, *K*_R_ resistance, hydraulic pressure, initial crack depth

## Abstract

The *K*_R_ resistance curve for hydraulic crack propagation in a concrete beam was determined and discussed. A semi-analytical method was introduced to calculate the hydraulic crack propagation in concrete. A series of concrete beams with various hydraulic pressures and initial crack depths were tested, and the hydraulic crack propagation in these beams was calculated. The calculated *P-CMOD* curves were first verified, and then the calculated *K*_R_ resistance curve for hydraulic crack propagation was determined. Based on the test results and calculation results, the following conclusions can be drawn: The proposed analysis method can accurately predict the hydraulic crack propagation process in concrete. The *K*_R_ resistance to hydraulic crack propagation in concrete decreases with the increase in hydraulic pressure but is less influenced by the initial crack depth of the test beams. In addition, the concrete beams collapse immediately under hydraulic fracturing once the *K*_Iw_ curve reaches the *K*_R_ resistance curve. This indicates that the failure of concrete structures under hydraulic fracturing occurs immediately once the driving force of crack propagation, dominated by the hydraulic pressure in the crack, becomes significant.

## 1. Introduction

Hydraulic fracturing is a severe problem in hydraulic engineering and is the main cause of serious leakage accidents in high concrete dams, deep water tunnels, and water conveyance tunnels [1,2,3]. The splitting load effect of hydraulic pressure in cracks has been recognized by common research, and the hydraulic pressure transition zone in cracks has been observed in experiments [4,5], which is related to the shape of the crack opening. Generally, due to the weak tensile performance of concrete and the inevitable temperature stress during concrete pouring, cracks often occur in concrete structures. The existence of these initial cracks can easily trigger the initiation of hydraulic fracturing and the propagation of hydraulic cracks in concrete structures. Therefore, studying the propagation of hydraulic cracks in concrete is of great importance.

The key to investigating the hydraulic crack propagation in concrete is describing the resistance of crack propagation. To analyze the resistance characteristics of crack propagation in metal materials, an R resistance curve was proposed [6,7]. However, the R resistance curve has limitations in analyzing the fracture resistance of solid materials like concrete. It cannot accurately describe the increase in fracture toughness in the crack propagation. To solve this problem, Reinhardt and Xu [8,9] proposed using the *K*_R_ resistance curve instead of the R resistance curve to describe the crack propagation resistance of concrete. The crack propagation resistance in concrete is divided into two parts in the *K*_R_ resistance curve: one is the inherent crack resistance of concrete material named as $K_{Ic}^{ini}$, and the other is the resistance induced by the cohesion distributed in the fictitious crack zone. A large number of concrete experiments [10,11,12,13] show that the *K*_R_ resistance curve has high reliability in describing the resistance of crack propagation in concrete.

The determination of the *K*_R_ resistance curve of concrete is related to the fracture parameters, including elastic modulus, initiation fracture toughness, and the tensile stress softening curve. Therefore, numerous hydraulic fracturing tests of concrete are necessary to study the resistance of hydraulic crack propagation in concrete. Bruhwiler and Saouma [14,15] conducted a series of hydraulic fracturing tests to study the influence of hydraulic pressure on the fracture energy of concrete. They recommended the compact tensile specimen as the test specimen and proposed and verified the test method. Xu and Wang [16,17] recommended improved hydraulic fracturing tests of concrete based on a large compact tensile specimen and calculated the double-K fracture toughness in their research. John P. and Lee [18] performed four-point bending tests on reinforced concrete beams within hydraulic pressure and discussed the loading force and strain performance of reinforced concrete beams. Wang and Jia [19,20] employed notched columnar concrete specimens to study the critical hydraulic pressure in the crack that break the test specimen. Chen and Du [21,22] developed a new sealing device to conduct the hydraulic fracturing tests of concrete specimens and discussed the hydraulic pressure distributions in the crack under different loading rates. Wang [23,24,25] performed hydraulic fracturing tests on the three-point bending concrete beams to investigate the effect of water pressure on the fracture parameters of concrete. In these hydraulic fracturing tests, all tested specimens were precracked and filled with pressurized water to simulate hydraulic cracks in concrete. In these tests, the distribution of hydraulic pressure in cracks and the fracture parameters of the concrete are the focus of scholars’ attention; however, the discussion on the resistance of hydraulic crack propagation in the concrete is rarely mentioned.

The main objective of this paper is to study the *K*_R_ resistance curve of the concrete subjected to different hydraulic pressures. A semi-analytical method is proposed to calculate the propagation of the mode-I crack within hydraulic pressure in the concrete based on the fictitious crack mode by combining the deformation coordination equation and the initiation fracture toughness criterion. Moreover, a series of three-point bending (TPB) concrete beams subjected to hydraulic fracturing are assessed to verify the validity of the proposed method. Additionally, the effects of hydraulic pressure and initial crack depth ratio on the *K*_R_ resistance and the hydraulic crack propagation in concrete are analyzed and discussed.

## 2. Analysis of Hydraulic Crack Propagation in Concrete Beam

### 2.1. Criterion of Hydraulic Crack Propagation in Concrete

**Figure 1:**
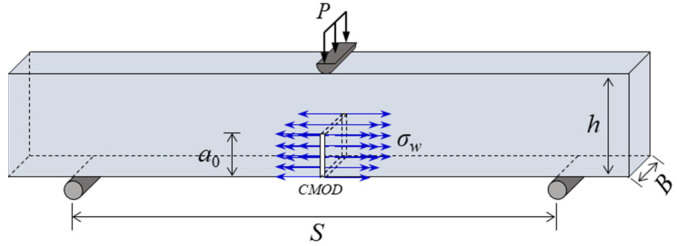
Analysis of hydraulic fracturing load of concrete beam.

Figure 1 shows the loading forces applied on a standard TPB notched concrete beam under hydraulic fracturing. The geometry of the beam is S in the span, h in the height, and B in the thickness, and the length of the initial crack in the middle of the beam is *a*_0_. Figure 1 shows external load *P* and hydraulic pressure $\sigma_{w}$ acting on the crack surface, which are the driving forces of the hydraulic crack propagation in concrete.

All forces applied on the concrete beam under hydraulic fracturing can be divided into three parts: external load P, internal hydraulic pressure $\sigma_{w}$, and cohesion in the crack σ. According to the superposition principle, the stress intensity factor (SIF) at the crack tip *K*_e_ can be evaluated using the following equation:

(1) $$K_{e}=K_{IP}+K_{Iw}-K_{I\sigma }$$

where $K_{e}$ is the SIF at the crack tip, $K_{IP}$ is the SIF induced by the loading force *P*, $K_{Iw}$ is the SIF induced by the hydraulic pressure in the crack, and $K_{I\sigma }$ is the SIF induced by the cohesive force in FPZ.

For a crack that continuously propagates in quasi-brittle materials, the SIF of the crack tip should satisfy the following equation:

(2) $$K_{e}=K_{Ic}^{ini}$$

where $K_{Ic}^{ini}$ is the initial fracture toughness of the materials. This crack propagation criterion has been proven by Wu and Dong [26,27] in a simulation of crack propagation in concrete. Therefore, the hydraulic crack propagation criterion can be proposed as follows:

(3) $$K_{IP}+K_{Iw}-K_{I\sigma }=K_{Ic}^{ini}$$

The SIF induced by loading force *P*, for the TPB beam with a crack length of *a*, can be calculated by the following formula [28]:

(4) $$K_{IP}=\frac{3PS\sqrt{a}}{2h^{2}B}F_{1}(\frac{a}{h})$$

(5) $$F_{1}(\frac{a}{h})=\frac{1.99-(\frac{a}{h})(1-\frac{a}{h})[2.15-3.93(\frac{a}{h})+2.7{\left(\frac{a}{h}\right)}^{2}]}{(1+\frac{2a}{h}){\left(1-\frac{a}{h}\right)}^{3/2}}$$

According to the fictitious crack model, hydraulic pressure $\sigma_{w}$ and cohesive force σ are considered to act on both sides of the fictitious crack zone. The SIFs induced by hydraulic pressure and cohesive force can be determined by Equation (6) [23] and Equation (7) [29], respectively.

(6) $$K_{Iw}=\int_{0}^{a}\frac{2\sigma_{w}(x)}{\sqrt{\pi a}}F_{2}(\frac{x}{a},\frac{a}{h})dx$$

(7) $$K_{I\sigma }=\int_{a_{0}}^{a}\frac{2\sigma (x)}{\sqrt{\pi a}}F_{2}(\frac{x}{a},\frac{a}{h})dx$$

(8) $$F_{2}(\frac{x}{a},\frac{a}{h})=\frac{3.52(1-\frac{x}{a})}{{\left(1-\frac{a}{h}\right)}^{3/2}}-\frac{4.35-5.28(\frac{x}{a})}{{\left(1-\frac{a}{h}\right)}^{1/2}}+\left\{\left.\frac{1.3-0.3{\left(\frac{x}{a}\right)}^{3/2}}{\sqrt{1-{\left(\frac{x}{a}\right)}^{2}}}+0.83-1.76(\frac{x}{a})\right\}\right.[1-(1-\frac{x}{a})(\frac{a}{h})]$$

**Figure 2:**
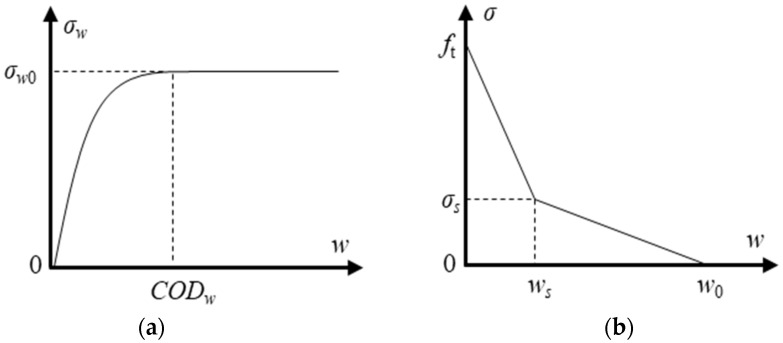
Hydraulic pressure-displacement and cohesive stress-displacement relationships. (**a**) Quadratic function of $\sigma_{w}-w$, (**b**) bilinear function of σ−w.

Previous researchers have shown that the hydraulic pressure and cohesive force applied on the crack surface can be described as quadratic and bilinear functions of crack width, respectively. The quadratic function relation of the hydraulic pressure $\sigma_{w}(x)$ and crack width *w*(*x*) shown in Figure 2a can be expressed as follows [23]:

(9) $$\sigma_{w}(x)=\left\{\begin{array}{c} A{\left(\frac{w(x)}{COD_{w}}\right)}^{2}\sigma_{w0}+B(\frac{w(x)}{COD_{w}})\sigma_{w0}\;\;\;,\;w(x)\leq COD_{w}\\ \;\;\;\;\sigma_{w0}\;\;\;\;\;\;\;,\;COD_{w}<w(x) \end{array}\right.$$

where *COD* is the crack opening displacement, *COD*_w_ is the critical crack width, and A and B are the fitting parameters of the relation between internal hydraulic pressure and crack width; *COD*_w_, A, and B can be obtained using a hydraulic fracture test of concrete beams [23].

Moreover, the bilinear function shown in Figure 2b can be employed to represent the softening tensile relationship of the cohesive stress in the fracture process zone (FPZ) of the crack. The relationship between the cohesive stress σ(*x*) and the crack width *w*(*x*) can be expressed as follows:

(10) $$\sigma (x)=\left\{\begin{array}{l} f_{t}-(f_{t}-\sigma_{s})w(x)/w_{s}\;\;\;\;\;0\leq w(x)\leq w_{s}\\ \sigma_{s}\left(w_{0}-w(x)\right)/\left(w_{0}-w_{s}\right)\;\;w_{s}<w(x)\leq w_{0}\\ 0\;\;\;\;\;\;\;\;\;\;\;\;w_{0}<w(x) \end{array}\right.$$

where $f_{t}$ is the tensile strength. The key parameters $w_{s}$, $w_{0}$, and $\sigma_{s}$ in Equation (10) can be obtained based on the fracture energy $G_{F}$ and tensile strength $f_{t}$ of concrete as follows:

(11) $$\left\{\begin{array}{l} w_{s}=0.8\;G_{F}/f_{t}\\ w_{0}=3.6\;G_{F}/f_{t}\\ \sigma_{s}=f_{t}/3 \end{array}\right.$$

### 2.2. Determination of Hydraulic Crack Width in Concrete

Equations (8) and (9) show that the determinations of the internal hydraulic pressure $\sigma_{w}(x)$ and the cohesive force σ(x) are related to the crack width w(x). For the concrete beam, the crack width, w, is produced by the external loading force, the hydraulic pressure $\sigma_{w}(x)$, and the cohesion σ(x) in the crack. Hence, the w(x) of the hydraulic crack can be mathematically written as follows:

(12) $$w(x)=w^{P}(x)+w^{\sigma_{w}}(x)-w^{\sigma }(x)$$

where w(x) is the crack width of the hydraulic crack at a distance of *x* to the crack mouth, $w^{P}(x)$ is the crack width of the hydraulic crack created by the external load, $w^{\sigma_{w}}(x)$ is the crack width of the hydraulic crack created by the hydraulic pressure in the crack, and $w^{\sigma }(x)$ is the crack width of the hydraulic crack created by the cohesion in the crack.

The crack width $w^{P}(x)$ created by the external force *P* can be obtained by the following formula:

(13) $$w^{P}(x)={\mathrm{CMOD}}^{p}{\left[{\left(1-\frac{x}{a}\right)}^{2}+(1.081-1.149\frac{a}{h})\frac{x}{a}(1-\frac{x}{a})\right]}^{1/2}$$

where $CMOD^{P}$ represents the crack mouth opening displacement of test concrete beams created by the external load *P*, which can be obtained by Equation (14) [26]:

(14) $${\mathrm{CMOD}}^{p}=\frac{24P\lambda }{EB}\left[0.76-2.28\lambda +3.87\lambda^{2}-2.04\lambda^{3}+\frac{0.66}{{\left(1-\lambda \right)}^{2}}\right]$$

where *E* is the Young’s modulus of the concrete beam, and λ=a/h.

For the hydraulic crack in the concrete, the internal forces applied on the crack surface consist of hydraulic pressure $w^{\sigma_{w}}(x)$ and cohesive force $w^{\sigma }(x)$. Thus, the crack width of the hydraulic crack can be obtained by the superposition of the crack width under a pair of concentrated loads using Castigliano’s theorem.

**Figure 3:**
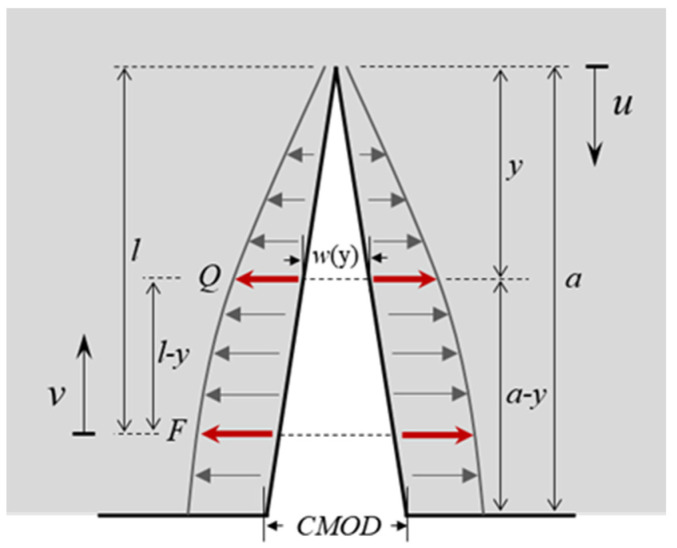
Estimation of hydraulic crack face separation by Castigliano’s theorem.

Figure 3 shows the crack width *w*(y) of the crack with a length subjected to a pair of concentrated loads, *F*, with a distance of *l* to the crack tip which can be given by [30].

(15) $$w(y)=\frac{2}{E}\int_{l-y}^{l}K_{F}(u){\left[\frac{\partial K_{Q}(u-l+y)}{\partial Q}\right]}_{Q=0}du$$

Here, *u* is the distance of the calculated point to the crack tip; *v* is the distance of the calculated point to the point of application of *F*; *Q* is a pair of fictitious concentrated forces in the crack with a distance of y to the tip; and *K*_F_ and *K*_Q_ are the SIFs created by *F* and *Q*, respectively. Exchanging the locations of *F* and *Q* in the crack, Equation (15) can be rewritten as follows:

(16) $$w(y)=\frac{2}{E}\int_{y-l}^{l}K_{F}(u-y+l){\left[\frac{\partial K_{Q}(u)}{\partial Q}\right]}_{Q=0}du$$

The internal stress applied in the hydraulic crack can be calculated as $\sigma_{c}(x)=\sigma_{w}(x)-\sigma (x)$. The internal stress can be divided into two parts: the stress distributed along the range of 0≤v≤x denoted as $\sigma_{1}(x)$ and the stress distributed along the range of x<v≤a denoted as $\sigma_{2}(x)$. Substituting $F=\sigma_{c}(v)\cdot \Delta v=[\sigma_{w}(v)-\sigma (v)]\cdot \Delta v$, Equations (5) and (6) into Equation (15), and integrating *v* along the range of 0≤v≤x, the crack width $w^{\sigma_{1}}(x)$ induced by $\sigma_{1}(x)$ can be calculated as follows:

(17) $$w^{\sigma_{1}}(x)=\frac{8}{\pi E}\int_{0}^{x}\int_{x}^{a}\frac{\sigma_{w}(v)-\sigma (v)}{u}F_{2}(\frac{v}{u},\frac{u}{h})F_{2}(\frac{x}{u},\frac{u}{h})dudv$$

Similarly, substituting $F=\sigma_{c}(v)\cdot \Delta v=[\sigma_{w}(v)-\sigma (v)]\cdot \Delta v$, Equations (5) and (6) into Equation (16), and changing the integration path of *v* to the range of x<v≤a, the crack width $w^{\sigma_{2}}(x)$ created by $\sigma_{2}(x)$ can be calculated as follows:

(18) $$w^{\sigma_{2}}(x)=\frac{8}{\pi E}\int_{x}^{a}\int_{v}^{a}\frac{\sigma_{w}(v)-\sigma (v)}{u}F_{2}(\frac{v}{u},\frac{u}{h})F_{2}(\frac{x}{u},\frac{u}{h})dudv$$

The crack width $w^{\sigma_{c}}(x)$ induced by internal forces applied in the crack can be obtained by Equation (19), which is a combination of Equations (17) and (18) as follows:

(19) $$w^{\sigma_{c}}(x)=w^{\sigma_{1}}(x)+w^{\sigma_{2}}(x)$$

Therefore, the crack width induced by the hydraulic pressure and the cohesive force can be obtained by substituting $w^{\sigma_{c}}(x)=w^{\sigma_{w}}(x)-w^{\sigma }(x)$ into Equation (19) as follows:

(20) $$w^{\sigma_{w}}(x)-w^{\sigma }(x)=\frac{8}{\pi E}\int_{x}^{a}\int_{v}^{a}\frac{\sigma_{w}(v)-\sigma (v)}{u}F_{2}(\frac{v}{u},\frac{u}{h})F_{2}(\frac{x}{u},\frac{u}{h})dudv$$

Crack width w(x) can be calculated by combining Equations (13) and (20).

If x = 0, w(0) represents the crack mouth opening displacement, *CMOD*, of the hydraulic crack and can be expressed as follows:

(21) $$CMOD=CMOD^{P}+CMOD^{w}-CMOD^{\sigma }$$

where $CMOD^{P}$ is the *CMOD* created by *P* which can be obtained by Equation (15), $CMOD^{w}$ is the *CMOD* created by the hydraulic pressure, and $\sigma_{w}(x)$ and $CMOD^{\sigma }$ represent the *CMOD* created by the cohesion σ(x). Therefore, the *CMOD* of the hydraulic crack in the concrete beam can be expressed as follows:

(22) $$\begin{array}{cc} CMOD= & \frac{24P\lambda }{EB}\left[0.76-2.28\lambda +3.87\lambda^{2}-2.04\lambda^{3}+\frac{0.66}{{\left(1-\lambda \right)}^{2}}\right]\\ & +\frac{8}{\pi E}\int_{0}^{a}\int_{v}^{a}\frac{\sigma_{w}(v)-\sigma (v)}{u}F_{2}(\frac{v}{u},\frac{u}{h})F_{2}(0,\frac{u}{h})dudv \end{array}$$

Based on the previous analysis, the *CMOD* can be obtained by calculating the deformation of the hydraulic crack induced by P, $\sigma_{w}(x)$, and σ(x). The solutions of $\sigma_{w}(x)$ and σ(x) are governed by *w*(*x*), whereas *w*(*x*) is governed by *CMOD* as follows:

(23) w(x)=N(x)⋅CMOD

where *N*(*x*) is the shape function of a crack in the TPB beam and relating to the *CMOD*, it can be expressed as follows:

(24) $$w(x)=CMOD{\left\{{\left(1-x/a\right)}^{2}+[1.081-1.149(a/h)][x/a-{\left(x/a\right)}^{2}]\right\}}^{1/2}$$

Hence, a correlation must exist between *CMOD*, *P*, and $\left[\sigma_{w}(x)-\sigma (x)\right]$ for testing a concrete TPB beam with a notch length of *a*. All parameters of the test beam can be determined when *CMOD* and *P* are known.

### 2.3. Calculation of Hydraulic Crack Propagation

**Figure 4:**
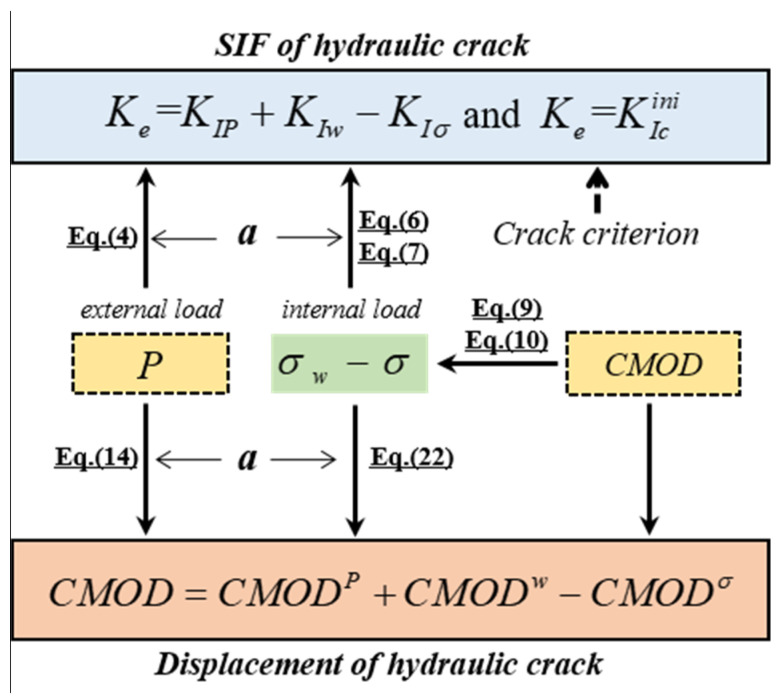
Relationships of the *P*, *CMOD*, and *K*_e_ of the hydraulic crack.

The previous analysis revealed that for a standard TPB beam with a notch length a, the calculation of the hydraulic crack propagation consists of three unknown quantities: loading forces, crack deformation, and the SIF of the crack tip. The solutions of crack deformations and the SIFs induced by cracks are governed by *CMOD*. A nonexplicit functional relationship exists between *P*, *CMOD*, and *K*_e_, as shown in Figure 4. Consequently, *CMOD* and *P* can be used as control parameters for calculating the hydraulic crack propagation. In the calculation model of hydraulic crack propagation, Δ*a*, Δ*CMOD*, and Δ*P* are the increased step lengths of *a*, *CMOD*, and *P*, respectively.

The specific computation procedures of the crack propagation for TPB concrete beams under hydraulic fracturing can be summarized as follows:

(1) Input the parameters required for the calculation, including the geometrical dimensions (span S, width B, height h, and initial notch length *a*_0_) of the test beam and the material parameters (Young’s modulus *E*, tensile strength *f*_t_, fracture energy *G_F_*, and initial fracturing toughness $K_{Ic}^{ini}$) of the concrete.
(2) Specify the expansion of the crack length increment, Δ*a = a +* Δ*a*, Δ*a* = 0.0001 m; employ the increment of crack mouth opening displacement, *CMOD* = *CMOD* + Δ*CMOD*, Δ*CMOD* = 0.00002m; apply the increment of external load, *P* = *P* + Δ*P*, Δ*P* = 0.0001 kN.
(3) Calculate $K_{IP}$, $K_{Iw}$, $K_{I\sigma }$, $CMOD^{P}$, $CMOD^{w}$, and $CMOD^{\sigma }$ by increasing *P* and CMOD until $K_{IP}+K_{Iw}-K_{I\sigma }=K_{Ic}^{ini}$ and $CMOD=CMOD^{P}+CMOD^{w}-CMOD^{\sigma }$ are satisfied. This iterative process is terminated until the increase in applied load *P* cannot satisfy the $K_{IP}+K_{Iw}-K_{I\sigma }=K_{Ic}^{ini}$, and the maximum load *P*_max_ and the critical crack mouth opening displacement *CMOD*_c_ are determined.
(4) Calculate $K_{IP}$, $K_{Iw}$, $K_{I\sigma }$, $CMOD^{P}$, $CMOD^{w}$, and $CMOD^{\sigma }$ by decreasing *P* and increasing *CMOD* until $K_{IP}+K_{Iw}-K_{I\sigma }=K_{Ic}^{ini}$ and $CMOD=CMOD^{P}+CMOD^{w}-CMOD^{\sigma }$ are satisfied. This iterative process is terminated when the crack tip reaches the boundary of the concrete. *P* < 0 is used in the calculation of satisfying $K_{IP}+K_{Iw}-K_{I\sigma }=K_{Ic}^{ini}$, but *P* = 0 stands in the calculation of satisfying $CMOD=CMOD^{P}+CMOD^{w}-CMOD^{\sigma }$.
(5) Output the calculated results of the characteristic curves of the hydraulic crack propagation process of the test concrete, including the *P-CMOD* curve, the *K*_R_ curve, the *K*_IP_ curve, and the *K*_Iw_ curve.

During the calculation, the applied vertical loading force *P*, the internal hydraulic pressure σ_w_, and the cohesive force σ, as well as the corresponding SIFs induced by these forces, such as *K*_IP_, *K*_Iw_, and *K*_Ic_, can be obtained in the iteration.

## 3. Hydraulic Fracturing Test of Concrete Beams

### 3.1. Experiment Program

**Figure 5:**
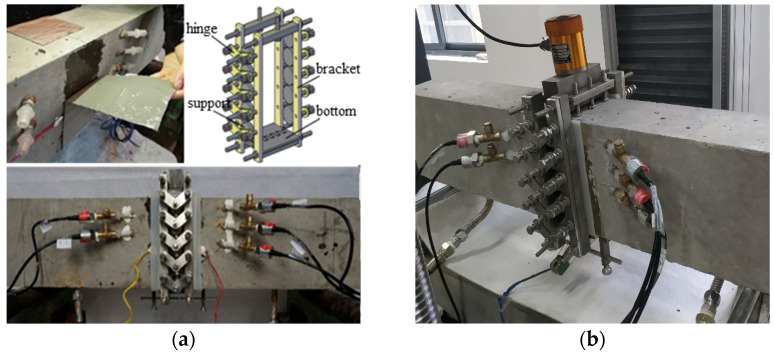
Hydraulic fracturing test of concrete beam. (**a**) Hydraulic test setup [24], (**b**) experimental setup.

In this experiment, 120 mm × 200 mm × 1000 mm molds were used to pour 30 concrete beams divided into 11 groups at one time. To create the crack in the beam, a 3 mm width steel plate was pre-embedded at the center of the timber molding plate before pouring. To create a hydraulic system in the crack, two metal pipes were pre-embedded at each side of the steel plate. Specimens stood 24 h before demolition, covered with cloth, and watered periodically in the laboratory for 28 days. The concrete beam was mixed from cement, natural river sands, and two levels of gravel aggregates. The Young’s modulus, compressive strength, and tensile strength of test concrete are 32.4 GPa, 27.9 MPa, and 3.12 MPa, respectively. The concrete composition is listed in Table 1. The arrangements of all test beams are listed in Table 2. The hydraulic fracturing test setup of the concrete beams is shown in Figure 5.

### 3.2. Experiment Results

**Figure 6:**
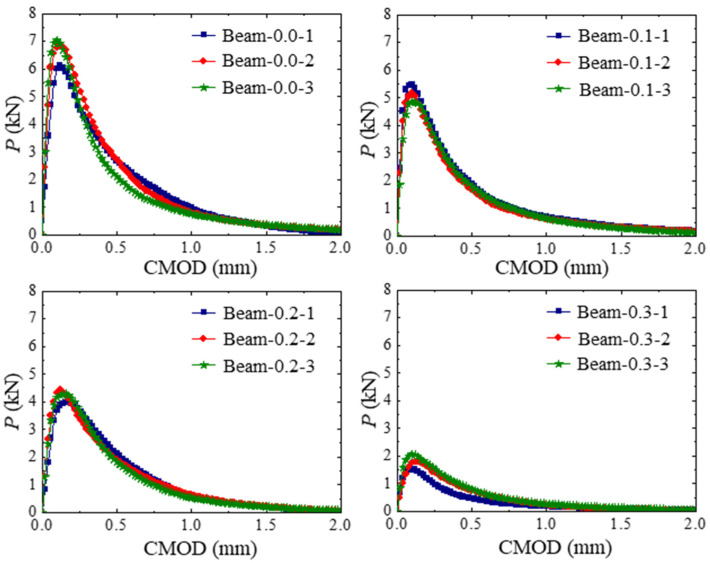
P-CMOD curves of concrete beams within different hydraulic pressures.

The P-CMOD curves of concrete beams within different hydraulic pressures are shown in Figure 6. Linear elastic deformation is observed at the early loading stages, and the loading force decreases to maintain the same *CMOD* with the increase in hydraulic pressure. By increasing the hydraulic pressure applied in the crack, the peak value of the loading force decreases. The fracture parameter results of the test concrete beams in Table 3 show that when the internal hydraulic pressure is increased, the initial load and maximum load are decreased.

(25) $$\left\{\begin{array}{l} w_{s}=CTOD_{c}\\ w_{0}=4.5CTOD_{c}\\ \sigma_{s}=f_{t}/3 \end{array}\right.$$

**Figure 7:**
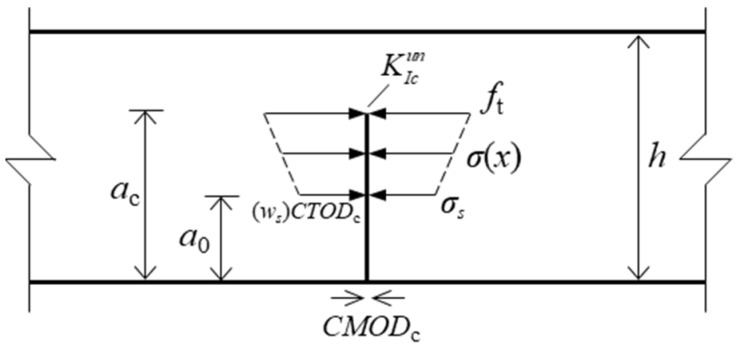
Cohesive stress applied at the crack surface when *a = a_c._*

To determine the softening curve parameters of the concrete under different hydraulic pressures, the crack tip opening displacement (*CTOD*) of the test beam was measured. When *a* = *a*_c_, the cohesive stress applied on the crack surface is shown in Figure 7, and the critical crack tip opening displacement (*CTOD*_c_) is equal to *w*_s_ based on reference [14]. Hence, the softening curve parameters calculated by Equation (11) can be translated to Equation (25). The parameters of the hydraulic pressure curve were obtained by fitting the internal hydraulic pressure data collected by the transducer installed at the end of the preset holes. The results of the fitting parameters of the hydraulic pressure curve and softening stress curve are listed in Table 4. As the applied hydraulic pressure increases from 0.0 MPa to 0.3 MPa, the peak load of test beams decreases by 60% and the cohesion fracture toughness decreases by 30%.

**Figure 8:**
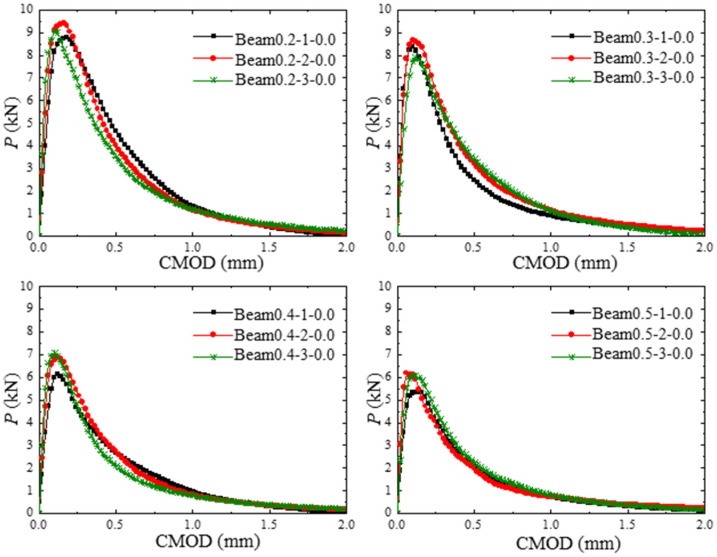
P-CMOD curves of test beams with different initial crack depths.

**Figure 9:**
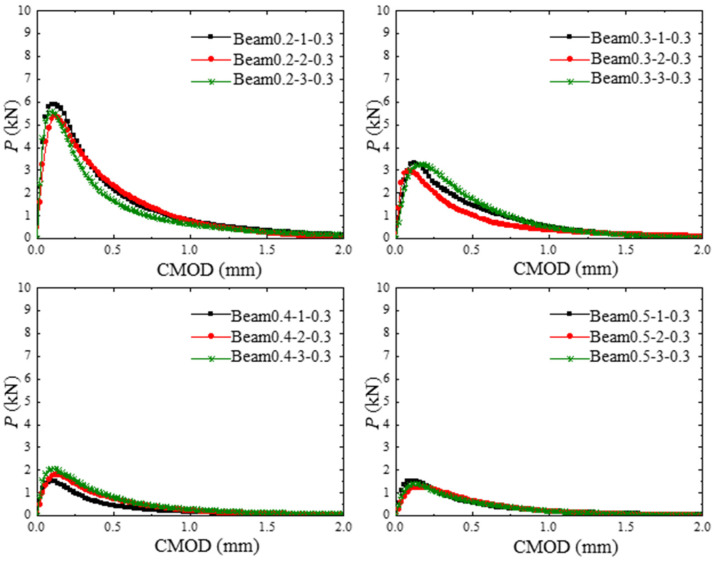
P-CMOD curves of test beams with different initial crack depths subjected to 0.3 MPa hydraulic pressure.

The P-CMOD curves of the concrete beams with different initial crack depth ratios under the hydraulic pressures of 0.0 and 0.3 MPa are shown in Figure 8 and Figure 9. The peak load of the test beam decreases with the increase in initial crack depth. When the hydraulic pressure applied in the crack increases from 0.0 MPa to 0.3 MPa, the cracking load and the maximum load decrease dramatically, as shown in Table 5, where $K_{\mathrm{Ic}}$ is the cohesive fracture toughness of the concrete. Moreover, the reduction in maximum load induced by hydraulic pressure increases with the increase in the initial crack depth ratio. In addition, the influence of the initial crack depth ratio on the fracture parameters under the same hydraulic pressure is slight.

## 4. Results and Discussion

### 4.1. Verification of Proposed Theoretical Model

**Figure 10:**
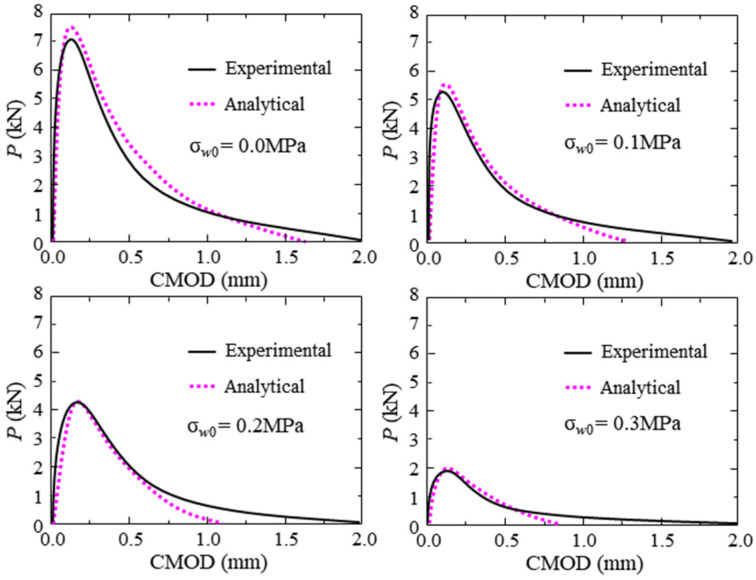
Comparison between analytical and experimental results within different hydraulic pressures.

**Figure 11:**
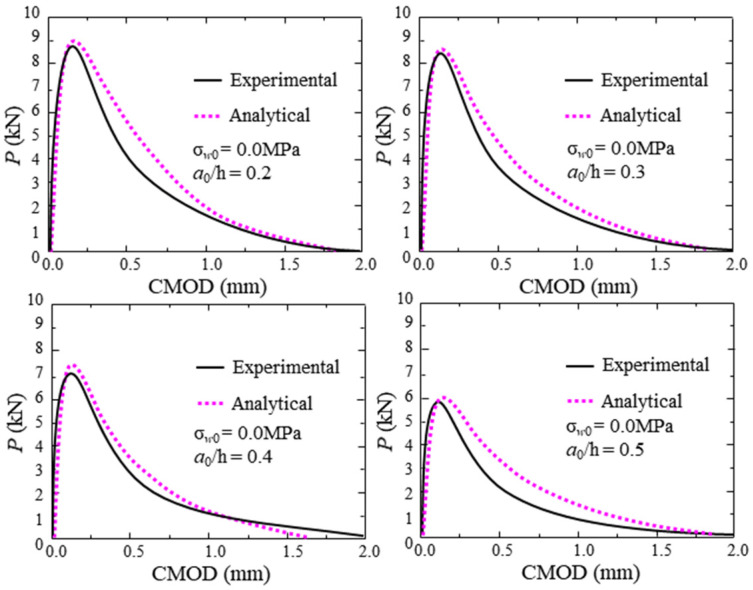
Comparison between analytical and experimental results without hydraulic pressure.

**Figure 12:**
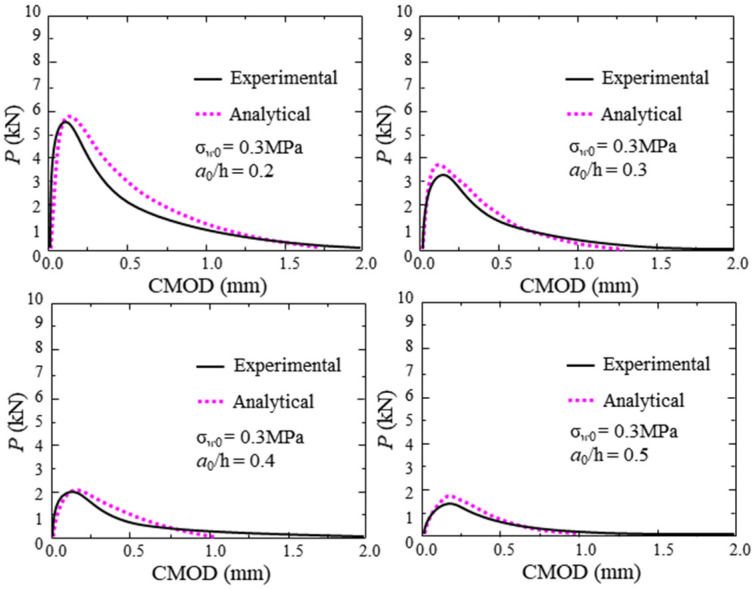
Comparison between analytical and experimental results with 0.3 MPa hydraulic pressure.

**Figure 13:**
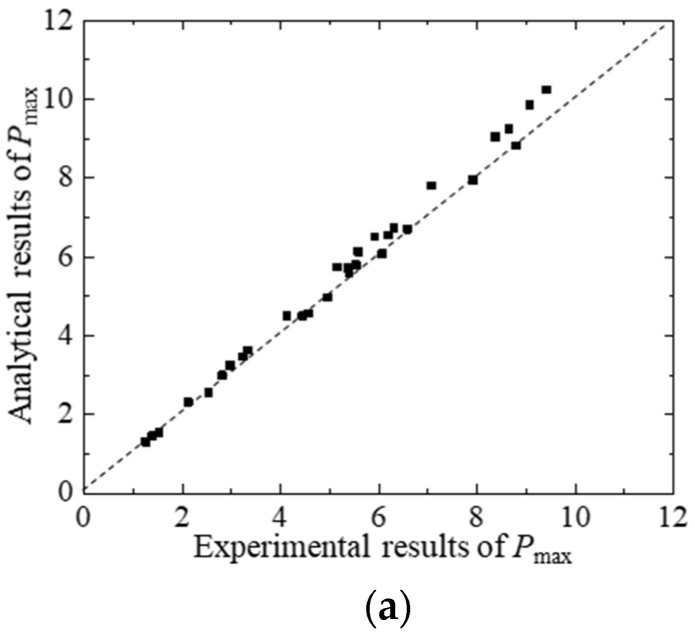

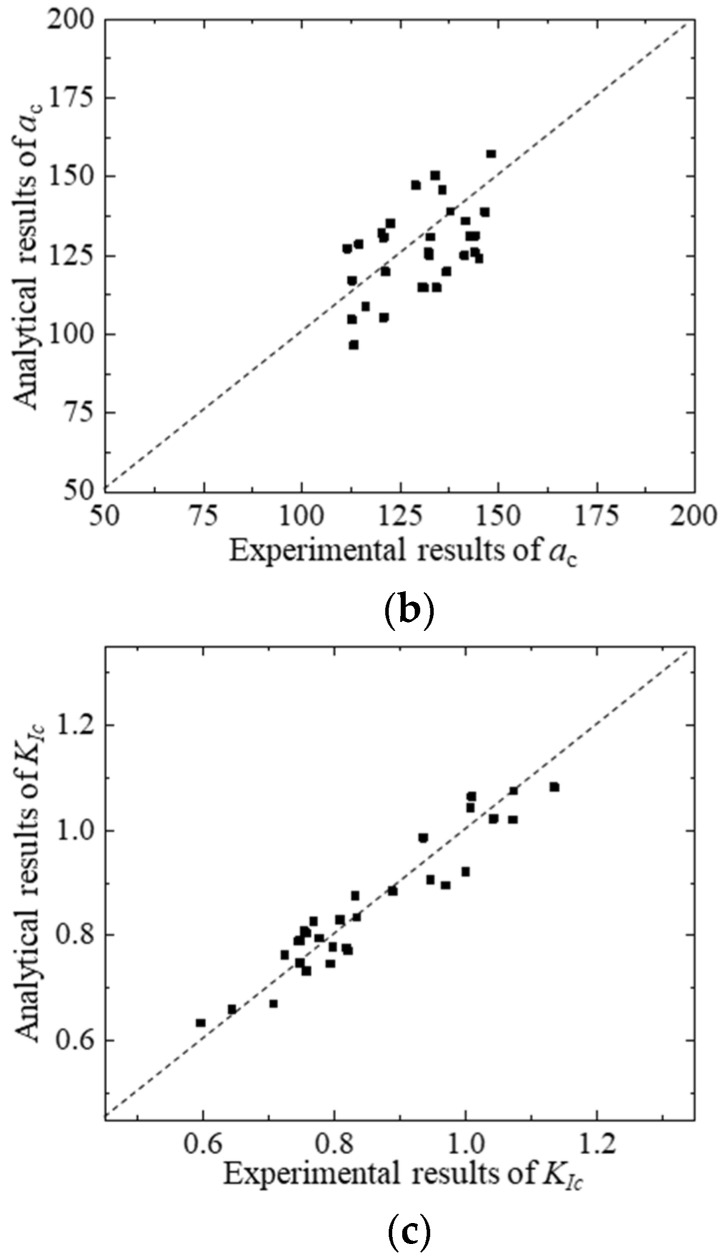
Comparison between theoretical and experimental results of test beams. (**a**) Comparison between analytical and experimental results of *P*_max_. (**b**) Comparison between analytical and experimental results of *a*_c_. (**c**) Comparison between analytical and experimental results of *K*_Ic_.

According to the material parameters and geometric parameters of the test concrete, the P-CMOD curve, *K*_R_-curve, and FPZ length can be calculated using the proposed analytical method. To consider the effect of the thickness of the clip gauge holder (H_0_ = 2.0 mm) and the self-weight on the experimental results, the calculated *P* and *CMOD* should be replaced by P − P*_w_* and *CMOD*(*a* + H_0_)/*a*, respectively. The comparisons between the analytical and experimental P-CMOD curves of test beams within different hydraulic pressures, which are displayed in Figure 10, show that the analytical predictions agree with the experimental results. Moreover, the predicted results of P-CMOD curves of test beams with different initial crack depths are described in Figure 11 and Figure 12. The results show good agreement between the predicted results and the experimental results. Furthermore, the predicted results of *P*_max_, *a*_c_, and *K*_Ic_ are compared with the measured results in Figure 13 and listed in Table 6. The predicted values agree with the measured values. Thus, the proposed analytical method has good applicability under different hydraulic pressures and diverse initial crack depth conditions.

### 4.2. Effect of Hydraulic Pressure on K_R_ Resistance Curve

**Figure 14:**
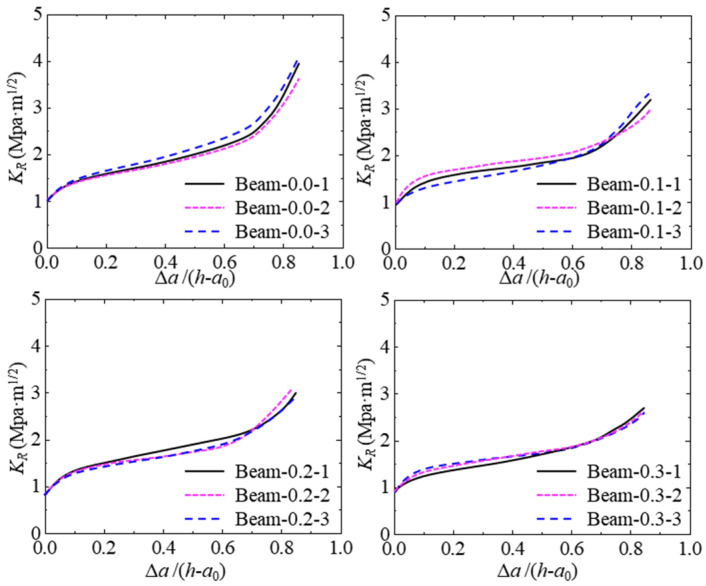
*K*_R_ curves for tested concrete beams with different hydraulic pressures.

Based on the proposed program, the theoretical P-CMOD curve of the test concrete beam can be calculated by inputting the geometric shape, elastic modulus, softening curve, and other parameters. After verification, the calculated *K*_R_ resistance curve of the test concrete beam can be obtained. The calculated *K*_R_ resistance curves of all tested beams are described in Figure 14. The shape difference among the *K*_R_ resistance curves under the same hydraulic pressure is quite small, which means the *K*_R_ resistance curve calculated by the proposed program can illustrate the inherent resistance of hydraulic crack propagation in concrete.

**Figure 15:**
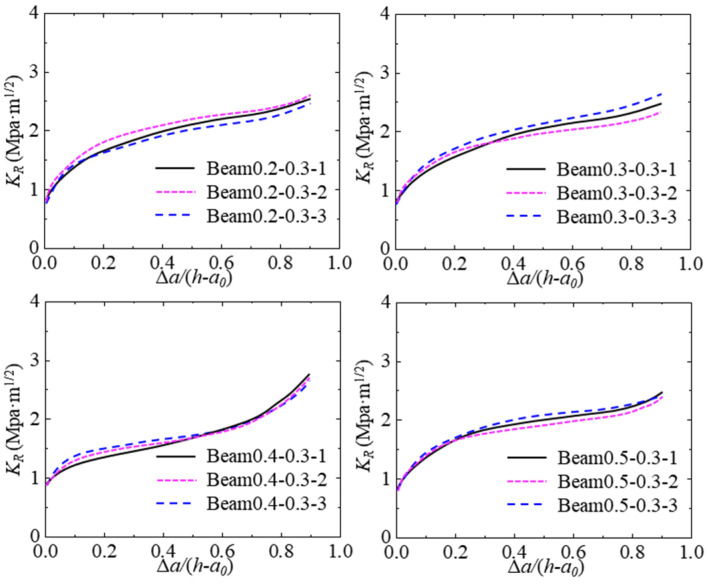
*K*_R_ curves for tested concrete beams with different initial crack lengths.

The *K*_R_ resistance curve of concrete increases when the crack propagation length increases, and the trend is to accelerate first and then slow down. Moreover, the *K*_R_ resistance is constant once it reaches the maximum value. Figure 15 shows the typical *K*_R_ resistance curves under different hydraulic pressures. The *K*_R_ resistance curves decrease with the increase in hydraulic pressure in the crack. Moreover, the starting points of all *K*_R_ resistance curves are close to one another, which represents that the initiation fracture toughness of the concrete within different hydraulic pressures is similar. In addition, the gaps between the *K*_R_ resistance curves under different hydraulic pressures are close. The degradation of *K*_R_ resistance induced by hydraulic pressure has a linear relation to the increase in hydraulic pressure.

### 4.3. Effect of Fracture Process Zone Length on K_R_ Resistance Curve

**Figure 16:**
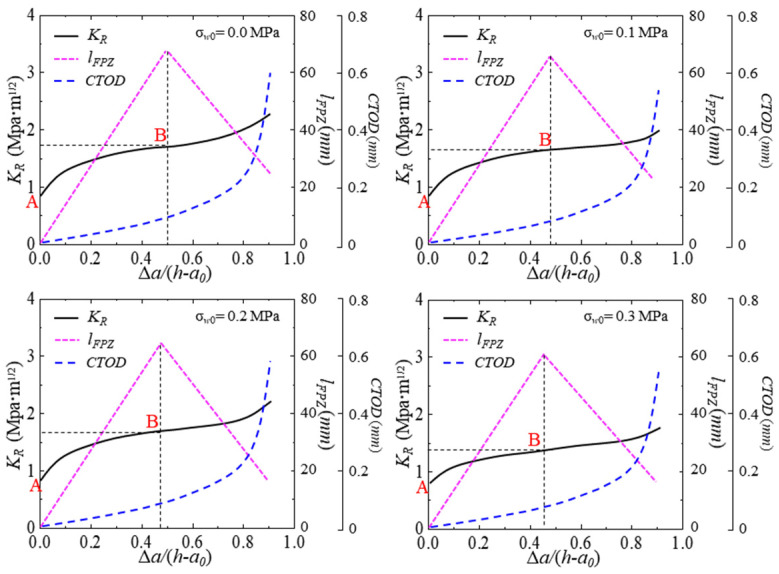
*K*_R_ curve, variation of *l*_FPZ_ and *CTOD* curve for test beams with different initial crack lengths. Point A is the initiation crack point and point B is the unstable crack propagation point.

To analyze the effect of hydraulic pressure on the *K*_R_ resistance curve more deeply, the FPZ length *l*_FPZ_ during the hydraulic crack propagation in concrete is recorded and illustrated in Figure 16. The *l*_FPZ_ is calculated as follows:

(26) $$\left\{\begin{array}{l} l_{FPZ}=a-a_{0}\;\;,\;CTOD\;\text{<}\;w_{0}\;\\ l_{FPZ}=a-a_{w_{0}}\;\;\;,\;CTOD\geq w_{0} \end{array}\right.$$

where $a_{w_{0}}$ is the critical crack length when *CTOD* = *w*_0_.

*l*_FPZ_ determines the cohesive fracture toughness $K_{Ic}\left(\Delta a\right)$. The effective integration interval is $K_{Ic}\left(\Delta a\right)$, so the influence of *l*_FPZ_ on the *K*_R_ curve needs to be studied. The calculation results of hydraulic crack propagation are analyzed below. Figure 16 describes the changes in the *K*_R_, *l*_FPZ_, and *CTOD* of test beams with crack propagation under different hydraulic pressures. The *l*_FPZ_ first increases and then decreases with the crack propagation. The *K*_R_ curve can be divided into two parts through points A and B, namely, the rising part and the approximate horizontal part. Point A represents that the crack just begins to expand, and the crack propagation resistance is equal to $K_{Ic}^{ini}$. Between points A and B represent stable and unstable crack propagation, and the *l*_FPZ_ increases gradually. The crack propagation resistance increases gradually with the crack propagation until it reaches the maximum value, that is, point B. At this time, the *l*_FPZ_ reaches the maximum, that is, a complete FPZ is formed. Subsequently, the value of *CTOD* exceeds the critical crack opening displacement *w*_0_, and *CTOD* clearly increases with the crack propagation, resulting in the formation of a new stress-free crack near the tip of the initial crack, and the *l*_FPZ_ decreases rapidly with the crack propagation. The decrease in *l*_FPZ_ leads to cohesive fracture toughness $K_{Ic}\left(\Delta a\right)$. The effective integral interval of $K_{Ic}\left(\Delta a\right)$ becomes smaller, resulting in no evident increase in crack propagation resistance. It shows that each specimen has a common feature, that is, when the peak load is reached, the stress intensity factor *K* and crack propagation resistance *K*_R_ are equal to the instability fracture toughness, whose corresponding abscissa is equal to $\left(a_{c}-a_{0}\right)/\left(h-a_{0}\right)$. In addition, when the value of Δ*a*/(h − *a*_0_) is between point A and point B, the crack expands stably. At this time, the driving force of crack development is less than the crack propagation resistance. When the value of Δ*a*/(h − *a*_0_) is greater than point B, the crack expands unsteadily. At this time, the driving force of crack development is greater than the resistance of crack propagation. Thus, the stability of crack propagation in the concrete can be expressed as follows:

- When $K\left(P,a\right)+K\left(\sigma_{w},a\right)<K_{R}\left(\Delta a\right)$, there is stable crack propagation;
- When $K\left(P,a\right)+K\left(\sigma_{w},a\right)\geq K_{R}\left(\Delta a\right)$, there is unstable crack propagation.

### 4.4. Analysis of Hydraulic Crack Propagation in Concrete

**Figure 17:**
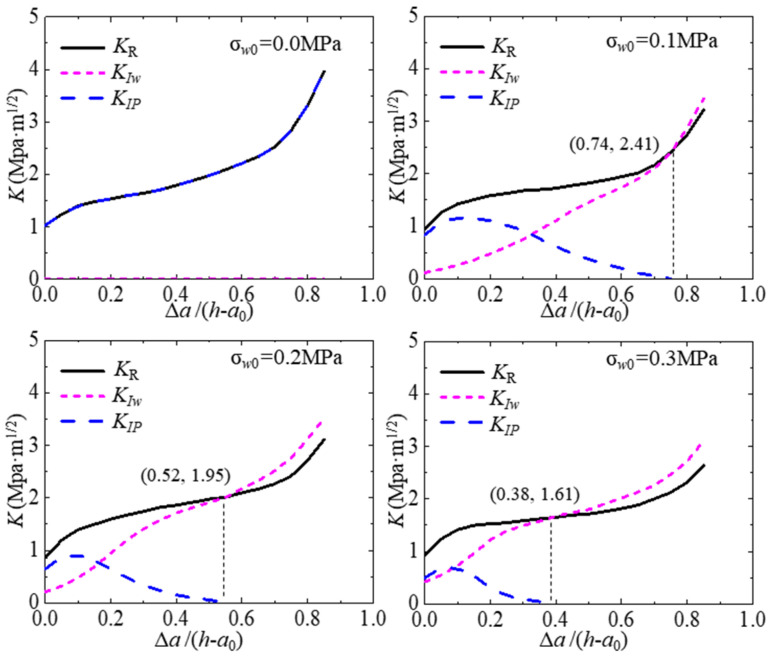
SIFs of hydraulic crack propagation in concrete.

A *K*_R_ resistance curve represents the concrete of the whole fracture process and can be used as the stability criterion of crack propagation in the whole fracture process. Before the stability analysis, the SIF curves in the crack propagation need to be calculated in advance. The load *P* and effective crack length *a* are substituted into the calculation formula to calculate the SIFs at the crack tip of the specimen at any loading time. Figure 17 shows the analysis of SIF curves and calculation results of typical specimens subjected to different hydraulic pressures of 0.0–0.3 MPa. The ratio of crack propagation length Δ*a* to specimen height *h* is shown in the figure below. Δ*a*/(*h* − *a*_0_) is the *x*-axis, and the *K*_R_ at the crack tip, the *K*_IP_ induced by *P*, and the *K*_Iw_ induced by the hydraulic pressure in the crack are the *y*-axis. Through this figure, the stability analysis of crack propagation of concrete structures in the whole fracture process can be conducted. It can be found that the *K*_R_ and *K*_Iw_ at the crack tip increase, but the *K*_IP_ first increases and then decreases to zero. This indicates that the driving force for the expansion of hydraulic fracture has shifted from load *P* to hydraulic pressure in the crack. Meanwhile, the hydraulic pressure in the crack increases with the increase in crack opening displacement. The hydraulic crack propagation in concrete is completely controlled by the hydraulic pressure in the crack. Thus, when $K\left(\sigma_{w},a\right)>K_{R}\left(\Delta a\right)$, the crack propagation in concrete accelerates until the specimen fails. This can be considered as the starting point of the hydraulic fracturing disaster effect of concrete structures. For simplicity, the stability analysis of hydraulic crack development can be expressed as follows:

- When $K\left(P,a\right)+K\left(\sigma_{w},a\right)<K_{R}\left(\Delta a\right)$, there is stable crack propagation;
- When $K\left(P,a\right)+K\left(\sigma_{w},a\right)\geq K_{R}\left(\Delta a\right)$, there is unstable crack propagation;
- When $K\left(\sigma_{w},a\right)\geq K_{R}\left(\Delta a\right)$, hydraulic crack propagation accelerates.

## 5. Conclusions

An analytical method is proposed to calculate the hydraulic crack propagation in concrete. This method is based on the fictitious crack model and the initial fracture toughness criterion. Subsequently, a series of concrete beams with varying initial crack depths and different hydraulic pressures within the cracks are tested to investigate hydraulic crack propagation in concrete. Based on this investigation, the analytical model is verified, and the hydraulic fracturing is analyzed. The following conclusions can be drawn:

(1) Through the deformation relationship and crack initiation criterion, an analytical method for the hydraulic fracture propagation in concrete is proposed. Based on this analytical method, given the geometry and material information of the test concrete beam, the P-CMOD curve, FPZ length, and SIF curves induced by different loads can be calculated. The accuracy of the proposed analytical method is verified by comparing the theoretical results with the experimental results.
(2) Increasing the internal hydraulic pressure decreases the carrying capacity of the test beam, the crack resistance of concrete, and the length of the FPZ of the hydraulic crack. As the applied hydraulic pressure increases from 0.0 MPa to 0.3 MPa, the peak load decreases by 60%, the cohesion fracture toughness decreases by 30%, and the maximum FPZ length decreases by 10%.
(3) Increasing the initial crack depth ratio decreases the initial cracking load and peak load of the test beam, but it has less influence on the crack resistance of concrete and the length of the FPZ of the hydraulic crack. As *a*_0_/h increases from 0.2 to 0.5, the initial cracking load and the peak load incur a substantial percentage reduction of 30%.
(4) The stability of crack propagation in concrete subjected to hydraulic fracturing decreases as the internal hydraulic pressure increases, but it is less affected by the initial crack depth ratio. Furthermore, crack propagation, which is primarily driven by hydraulic pressure, occurs earlier with the increase in internal hydraulic pressure. The values of Δ*a*/(h − *a*_0_) at the collapse of the concrete beam under hydraulic pressures of 0.1, 0.2, and 0.3 MPa are 0.74, 0.52, and 0.38, respectively.
(5) In this analytical method for hydraulic crack propagation in concrete, the softening curve parameters are determined based on the measured *CTOD*_c_, which may compromise the computational accuracy of the proposed analysis model. Consequently, a more reliable theoretical approach to determining softening curve parameters using this method requires further investigation.

In the future, the softening curve parameters of concrete under different hydraulic pressures will be studied, and the influence of hydraulic pressure on the softening constitutive model will be analyzed through different hydraulic pressure tests to determine the influence of hydraulic pressure on the softening curve parameters.

## Figures and Tables

**Table 1:** Mixture proportion of concrete (kg/m^3^).

Water	Cement	Sand	Gravel (5–12.5 mm)	Gravel (12.5–25 mm)
182	350	761	614	538

**Table 2:** Number of test concrete beams.

Beams	*a*_0_ (mm)	*a*_0_/*h*	σ*_w_*_0_ (MPa)	Amounts	Sources
Beam-0.0-X	80	0.4	0.0	3	Ref. [23]
Beam-0.1-X	80	0.4	0.1	3	
Beam-0.2-X	80	0.4	0.2	3	
Beam-0.3-X	80	0.4	0.3	3	
Beam0.2-X-0.0	40	0.2	0.0	3	This work
Beam0.3-X-0.0	60	0.3	0.0	3	
Beam0.4-X-0.0	80	0.4	0.0	3	
Beam0.5-X-0.0	100	0.5	0.0	3	
Beam0.2-X-0.3	40	0.2	0.3	3	
Beam0.3-X-0.3	60	0.3	0.3	3	
Beam0.4-X-0.3	80	0.4	0.3	3	
Beam0.5-X-0.3	100	0.5	0.3	3	

Here, X = 1, 2, and 3 represent the different numbers of test beams.

**Table 3:** Experimental results of fracture parameters.

Number	*a*_c_ (mm)	*P_ini_* (kN)	*P*_max_ (kN)	Fracture Toughness (MPa·m^1/2^)	
				$K_{Ic}^{ini}$	$K_{Ic}^{un}$
TPB-0.0-1	135.7	3.69	6.31	0.883	1.714
TPB-0.0-2	137.7	3.82	7.08	0.914	1.923
TPB-0.0-3	133.8	3.56	6.58	0.852	1.787
Average	135.7	3.69	6.66	0.883	1.808
TPB-0.1-1	141.5	2.58	4.96	0.854	1.601
TPB-0.1-2	132.7	2.83	5.53	0.922	1.756
TPB-0.1-3	136.8	2.76	5.16	0.903	1.657
Average	137.0	2.72	5.22	0.893	1.671
TPB-0.2-1	134.2	2.01	4.44	0.939	1.715
TPB-0.2-2	130.5	2.12	4.56	0.974	1.742
TPB-0.2-3	141.3	1.82	4.13	0.878	1.623
Average	135.3	1.98	4.38	0.93	1.693
TPB-0.3-1	132.3	1.42	2.12	0.789	1.385
TPB-0.3-2	130.9	1.31	2.81	0.757	1.575
TPB-0.3-3	132.2	1.15	2.53	0.745	1.502
Average	131.8	1.29	2.49	0.764	1.487

**Table 4:** Fitting parameters of the hydraulic pressure curve and softening stress curve.

Number	σ*_w_*_0_ (MPa)	Hydraulic Pressure Curve			Softening Stress Curve			
		A	B	*COD_w_* (μm)	$f_{t}$ (MPa)	$\sigma_{s}$ (MPa)	$w_{s}$ (μm)	$w_{0}$ (μm)
TPB-0.1-1	0.1	−1.21	2.07	81	3.12	1.04	32.45	146.03
TPB-0.1-2		−0.76	1.84	76	3.12	1.04	32.01	144.05
TPB-0.1-3		−0.94	1.98	104	3.12	1.04	31.42	141.39
Average		−0.97	1.96	87	3.12	1.04	31.96	143.82
TPB-0.2-1	0.2	−1.53	2.55	60	3.12	1.04	28.49	128.21
TPB-0.2-2		−1.21	2.23	76	3.12	1.04	28.67	129.02
TPB-0.2-3		−0.82	1.91	67	3.12	1.04	28.55	128.48
Average		−1.18	2.23	68	3.12	1.04	28.57	128.57
TPB-0.3-1	0.3	−0.94	2.01	51	3.12	1.04	27.99	125.96
TPB-0.3-2		−1.55	2.56	57	3.12	1.04	28.01	126.05
TPB-0.3-3		−1.13	2.15	60	3.12	1.04	27.49	123.71
Average		−1.21	2.24	56	3.12	1.04	27.83	125.24

**Table 5:** Experimental results of fracture parameters with different initial crack depths.

Number	*a*_c_ (mm)	*P*_ini_ (kN)	*P*_max_ (kN)	Fracture Toughness (MPa·m^1/2^)		
				$K_{\mathrm{Ic}}^{ini}$	$K_{\mathrm{Ic}}^{un}$	$K_{\mathrm{Ic}}$
TPB0.2-1-0.0	114.5	5.13	8.78	0.932	1.878	0.946
TPB0.2-2-0.0	112.7	5.08	9.41	0.917	2.042	1.135
TPB0.2-3-0.0	116.2	4.91	9.07	0.891	1.891	1.000
Average	114.5	5.03	9.08	0.913	1.937	1.038
TPB0.3-1-0.0	121.2	4.89	8.37	0.953	1.995	1.042
TPB0.3-2-0.0	120.8	4.67	8.65	0.911	1.983	1.072
TPB0.3-3-0.0	122.5	4.28	7.91	0.945	1.942	1.007
Average	121.2	4.61	8.31	0.933	1.973	1.042
TPB0.4-1-0.0	135.7	3.69	6.31	0.883	1.714	0.831
TPB0.4-2-0.0	137.7	3.82	7.08	0.914	1.923	1.009
TPB0.4-3-0.0	133.8	3.56	6.58	0.852	1.787	0.935
Average	135.7	3.69	6.66	0.883	1.808	0.925
TPB0.5-1-0.0	146.5	3.13	5.36	0.914	1.792	0.888
TPB0.5-2-0.0	148.1	3.34	6.19	0.909	1.982	1.073
TPB0.5-3-0.0	145.1	3.28	6.07	0.871	1.841	0.970
Average	146.5	3.25	5.87	0.891	1.874	0.983
TPB0.2-1-0.3	111.5	4.23	5.91	0.807	1.554	0.747
TPB0.2-2-0.3	113.3	3.86	5.39	0.755	1.512	0.757
TPB0.2-3-0.3	112.7	3.98	5.58	0.785	1.593	0.808
Average	112.5	4.02	5.62	0.781	1.554	0.773
TPB0.3-1-0.3	128.9	2.38	3.32	0.801	1.444	0.643
TPB0.3-2-0.3	120.3	2.13	2.97	0.736	1.556	0.820
TPB0.3-3-0.3	120.8	2.32	3.24	0.728	1.525	0.797
Average	123.7	2.27	3.18	0.759	1.520	0.761
TPB0.4-1-0.3	132.3	1.42	2.12	0.789	1.385	0.596
TPB0.4-2-0.3	130.9	1.31	2.81	0.757	1.575	0.818
TPB0.4-3-0.3	132.2	1.15	2.53	0.745	1.502	0.757
Average	131.8	1.29	2.49	0.764	1.487	0.723
TPB0.5-1-0.3	143.9	1.088	1.52	0.792	1.516	0.724
TPB0.5-2-0.3	142.7	0.895	1.25	0.736	1.529	0.793
TPB0.5-3-0.3	143.9	0.988	1.38	0.801	1.508	0.707
Average	143.6	0.988	1.38	0.773	1.518	0.745

**Table 6:** Comparison of experimental and calculated results.

Number		*a*_c_ (mm)	*P_ini_* (kN)	*P*_max_ (kN)	Fracture Toughness (MPa·m^1/2^)	
					$K_{Ic}^{ini}$	$K_{Ic}^{un}$
TPB-0.0-X	Experiment	135.7	3.69	6.66	0.883	1.808
	Calculation	135.9	3.62	7.36	0.831	1.925
	Error	0.1%	1.9%	9.5%	6.25%	6.1%
TPB-0.1-X	Experiment	137.0	2.72	5.22	0.893	1.671
	Calculation	138.5	2.61	5.63	0.832	1.754
	Error	1.1%	4.2%	7.3%	7.3%	4.7%
TPB-0.2-X	Experiment	135.3	1.98	4.38	0.93	1.693
	Calculation	135.0	1.82	4.37	0.88	1.689
	Error	0.2%	8.8%	0.2%	5.7%	0.2%
TPB-0.3-X	Experiment	131.8	1.29	2.09	0.764	1.487
	Calculation	130.9	1.22	2.10	0.732	1.443
	Error	0.7%	5.7%	0.5%	4.4%	3.0%
TPB0.2-X-0.0	Experiment	114.5	5.03	9.08	0.913	1.937
	Calculation	114.0	5.00	9.10	0.909	1.989
	Error	0.4%	0.6%	0.2%	0.4%	2.6%
TPB0.3-X-0.0	Experiment	121.2	4.61	8.31	0.933	1.973
	Calculation	120.8	4.58	8.46	0.920	1.987
	Error	0.3%	0.6%	1.8%	1.4%	0.7%
TPB0.4-X-0.0	Experiment	135.7	3.69	7.06	0.883	1.808
	Calculation	134.2	3.63	7.53	0.868	1.897
	Error	1.1%	1.7%	6.2%	1.7%	4.7%
TPB0.5-X-0.0	Experiment	146.5	3.25	5.87	0.891	1.874
	Calculation	140.9	3.28	5.99	0.887	1.901
	Error	4.0%	0.9%	2.0%	0.5%	1.4%
TPB0.2-X-0.3	Experiment	112.5	4.02	5.62	0.781	1.554
	Calculation	112.7	3.98	5.68	0.780	1.567
	Error	0.2%	1.0%	1.1%	0.1%	0.8%
TPB0.3-X-0.3	Experiment	123.7	2.27	3.18	0.759	1.520
	Calculation	124.0	2.20	3.21	0.750	1.531
	Error	0.2%	3.2%	0.9%	1.2%	0.7%
TPB0.4-X-0.3	Experiment	131.8	1.29	2.49	0.764	1.487
	Calculation	130.9	1.27	2.58	0.759	1.499
	Error	0.7%	1.6%	3.5%	0.7%	0.8%
TPB0.5-X-0.3	Experiment	143.6	0.988	1.38	0.773	1.518
	Calculation	142.8	0.972	1.45	0.780	1.529
	Error	0.6%	1.6%	4.8%	0.9%	0.7%

## Data Availability

Data are unavailable due to privacy.
